# SOX30 Overexpression Reflects Tumor Invasive Degree, Lymph Node Metastasis and Predicts Better Survival in Colorectal Cancer Patients: A Long-Term Follow-Up Cohort Study

**DOI:** 10.3389/fsurg.2022.898952

**Published:** 2022-06-28

**Authors:** Chao Li, Pengfei Li, Lulu Yu, Qingsen Sun, Bin Gu, Yanhua Sun, Liansheng Sun

**Affiliations:** ^1^ Department of Gastrointestinal Surgery, Cangzhou People’s Hospital, Cangzhou, China; ^2^ Department of Clinical Laboratory, Cangzhou People’s Hospital, Cangzhou, China

**Keywords:** sex-determining region Y-box containing gene 30, colorectal cancer, clinical characteristics, survival, disease progression

## Abstract

**Aims:**

Sex-determining region Y-box containing gene 30 (SOX30) takes part in the progression of several cancers, while its clinical engagement in colorectal cancer (CRC) is obscure. Therefore, this study aimed to explore the association of SOX30 with clinicopathological features and prognosis in CRC patients.

**Methods:**

Tumor and adjacent noncancerous specimens of 195 CRC patients who received resection were acquired. Furthermore, an immunohistochemistry assay was performed to detect SOX30 protein expression in these specimens; meanwhile, SOX30 mRNA expression was determined by reverse transcription-quantitative polymerase chain reaction assay in 95 out of 195 specimens. Moreover, clinical characteristics and survival data (follow-up duration median (range): 71.0 (7.0-95.0) months) of CRC patients were gathered.

**Results:**

SOX30 protein and mRNA expressions were both decreased in CRC tumor tissue compared to adjacent tissue (both *P *< 0.001). Furthermore, a negative correlation was found in tumor SOX30 protein expression with tumor size (*P *= 0.049), lymph node (LYN) metastasis (*P *= 0.018), T stage (*P *= 0.001), N stage (*P *= 0.034), and TNM stage (*P *= 0.001); tumor SOX30 mRNA expression was also negatively correlated with LYN metastasis (*P *= 0.001), T stage (*P *= 0.019), N stage (*P *= 0.004), and TNM stage (*P *< 0.001). Furthermore, tumor SOX30 protein expression was positively correlated with overall survival (OS) (*P *= 0.017), while tumor SOX30 mRNA expression was not correlated with OS (*P *= 0.070). Multivariate Cox’s regression analysis illustrated that tumor SOX30 protein high expression was an independent factor for favorable OS (hazard ratio: 0.525, *P *= 0.034).

**Conclusions:**

SOX30 has potential as a biomarker for the progression and prognostication of CRC, which might improve the management of CRC.

## Introduction

Colorectal cancer (CRC) is one of the most common cancers and the major cause of cancerous mortality globally (1–3). According to the WHO cancer research center GLOBOCAN project, the morbidity and mortality of CRC worldwide in 2018 are approximately 1.8 million and 88 thousand, respectively (4). Generally, the treatments of CRC include resection, radiotherapy, chemotherapy combinations, and immunotherapy (5). Although great progress in the treatments of CRC, unfavorable long-term survival is still an obstinate problem in CRC patients (1, 2, 5). In consideration that CRC is still prevalent cancer with an unsatisfied cure rate and elevated recurrence, the exploration of prognostic factors to improve CRC management is crucial (1, 5).

Sex-determining region Y-box (SOX) transcription factors have been illustrated to be involved in tumor progression, such as tumorigenesis and metastasis (6). Among famously identified SOX transcription factors, SOX containing gene 30 (SOX30) is considered to be a suppressor of a variety of cancers, including hepatocellular carcinoma, lung cancer, ovarian cancer, etc. (7–12). For instance, SOX30 is able to repress tumor metastasis in lung cancer by regulating the Wnt signaling pathway (8); SOX30 also plays an anti-metastatic role in ovarian cancer through modulating the epithelial-mesenchymal transition (EMT) process (9); it has been illustrated that SOX30 has the capacity of inhibiting proliferation and invasion but promoting apoptosis of T24 and 5,673 cells to suppress the progression of bladder cancer (12). Besides, SOX30 is regarded as a prognostic biomarker in terminal ovarian cancer patients (9); elevated SOX30 expression is regarded as a predictor of better survival among non-small cell lung cancer patients (10). On the basis of the above-mentioned data, we speculated that SOX30 could show an association with the progression and prognosis of CRC, while the clinical value of SOX30 in CRC remains unclear.

Therefore, the purpose of this present study was to explore the correlation of SOX30 expression measured by both immunohistochemistry (IHC) and reverse transcription-quantitative polymerase chain reaction (RT-qPCR) with clinicopathological features and long-term prognosis in CRC patients.

## Methods

### Patients

This retrospective study reviewed 195 CRC patients treated in the hospital between January 2013 and December 2017. The screening criteria for all subjects were: (i) diagnosed as primary CRC in accord with the European guidelines for colorectal cancer diagnosis (13); (ii) received tumor resections; (iii) had complete clinical characteristics and follow-up data; (iv) had available CRC specimens and paired adjacent noncancerous specimens to perform IHC assay. The exclusion criteria included: (i) diagnosed as relapsed CRC or secondary CRC at first admission; (ii) complicated with other carcinomas or malignancies at the time of diagnosis; (iii) had a history of chemotherapy, radiotherapy, or targeted therapy before resection. This study was approved by the Institutional Review Board of Cangzhou People's Hospital (ethics approval number: 20201127-2), and all patients or their statutory guardians signed the informed consent.

### Collection of Data and Specimens

By reviewing the medical documents, the clinical characteristics were collected, including age, gender, pathological grade, tumor size, lymph node (LYN) status, and TNM stage. Besides, overall survival (OS) data of all patients were also gathered, and the last follow-up date was February 2021. For specimens, CRC and paired adjacent noncancerous specimens of 195 patients, which were fixed in formalin and embedded in paraffin (FFEP), were collected to assess the SOX30 protein expression. Among 195 patients, 95 pairs of cancerous and adjacent noncancerous specimens frozen in liquid nitrogen were also collected to evaluate the SOX30 mRNA expression.

### SOX30 Protein Expression Detection

The SOX30 protein expression was assessed by IHC assay with 195 pairs of cancerous and adjacent tissue specimens. The rabbit anti-SOX30 polyclonal antibody (Thermo, Waltham, Massachusetts, USA) with 1:50 dilution was applied as the primary antibody, and the goat anti-rabbit IgG (H + L) (Thermo, Waltham, Massachusetts, USA) with 1:2,000 dilution was used as the secondary antibody. The process of the IHC assay was identical to the previous study (11). The assessment of the SOX30 protein expression was performed with a semi-quantitative scoring method as previously described (14). In brief, IHC staining results were observed by a light microscope. The intensity score of IHC staining was 0–3, while the density score of IHC staining was 0–4. The product of the intensity score and the density score generated a final IHC score, ranging from 0 to 12. The IHC score was evaluated by two pathologists in a blind manner. If the two pathologists gave different scores for the same sample, then the final IHC score of this sample was the mean value of the two IHC scores given by the two pathologists.

Western blot was conducted to verify the antibody against SOX30. After extracted by RIPA (Thermo, Waltham, Massachusetts, USA) and quantified by BCA kit (Beyotime, China), the protein was separated by sodium dodecyl sulfate polyacrylamide gel electrophoresis and transferred to polyvinylidene difluoride membranes (Beyotime, China). Then the membranes were incubated with primary antibodies (diluted at 1:2,000) at 4°C overnight, followed by secondary antibody incubation for 1 h at room temperature. After that, the brands were visualized by ECL kit (Yeason, China).

### RT-qPCR Assay

After the collection of 95 paired frozen specimens, the RT-qPCR assay was used to evaluate the expression of SOX30 mRNA. Briefly, total RNA was extracted by TRIzol™ Reagent (Invitrogen, Carlsbad, California, USA) and reversely transcribed by iScript™ cDNA Synthesis Kit (Bio-Rad, Hercules, California, USA). Afterwards, qPCR was performed by QuantiNova SYBR Green PCR Kit (Qiagen, Duesseldorf, Nordrhein-Westfalen, Germany). β-actin was used as an internal reference. Moreover, the relative expression of SOX30 mRNA was calculated by the 2^−ΔΔCt^ method. The PCR primer sequences were as follows: SOX30 forward: 5′-CCAAGCCCTGTCACACTTTT-3′ and reverse: 5′-AATCCTGTTGGCGCTCTCTA-3′; β-actin forward: 5′-CAATGACCCCTTCATTGACC-3′ and reverse: 5′-GACAAGCTTCCCGTTCTCAG-3′.

### Public Data for Prognostic Verification

We collected publicly available transcriptome data from two public databases to further verify the correlation of SOX30 expression with survival among CRC patients. A total of 597 CRC patients’ SOX30 RNA Fragments Per Kilobase Million (FPKM) data were obtained from The Human Protein Atlas (derived from The Cancer Genome Atlas (TCGA)) (available at https://www.proteinatlas.org/ENSG00000039600-SOX30/pathology/colorectal+cancer). Besides, a total of 90 CRC patients’ SOX30 Transcripts Per Million (TPM) data were obtained from GEPIA (available at http://gepia.cancer-pku.cn/detail.php?gene = SOX30###).

### Statistical Analysis

SPSS V.19.0 software (IBM Corp., Armonk, New York, USA) was used for statistical analysis and GraphPad Prism 7.02 (GraphPad Software Inc., San Diego, California, USA) was used for graph making. Paired-samples *t* test and Wilcoxon signed-rank test were applied to compare the SOX30 expression between CRC and paired adjacent noncancerous specimens. For correlation analysis of SOX30 with other variables, the SOX30 protein expression was classified as low expression (IHC score ≤3) and high expression (IHC score >3), respectively; and the median mRNA expression (0.392) in tumor tissue was used to classify the SOX30 mRNA low expression (≤0.392) and SOX30 mRNA high expression (>0.392), respectively. The correlations of the two variables were evaluated by the Chi-square test and the Mantel-Haenszel Chi-square test. Correlations between the SOX30 expression and accumulating OS were evaluated by Kaplan-Meier curves and log-rank test. Prognostic factors were determined by Cox’s proportional hazard regression model analysis. Statistical significance was concluded if a *P*-value <0.05 was presented in the corresponding analysis.

## Results

### Clinical Characteristics of CRC Patients

Among 195 CRC patients, the mean age was 65.7 ± 10.3 years, meanwhile, there were 118 (60.5%) males and 77 (39.5%) females. Regarding patients with different pathological grades, there existed 27 (13.9%) patients with grade I, 135 (69.2%) patients with grade II, and 33 (16.9%) patients with grade III. In addition, the median tumor size was 4.5 (3.5–5.0) cm in patients. With respect to LYN metastasis, 126 (64.6%) patients had LYN metastasis and 69 (35.4%) patients had no LYN metastasis. In terms of T stages, there were 5 (2.6%) patients with T1, 23 (11.8%) patients with T2, 165 (84.6%) patients with T3, and 2 (1.0%) patients with T4. Regarding N stages, there existed 121 (62.1%), 50 (25.6%), and 24 (12.3%) patients with N0, N1, and N2 accordingly. Concerning TNM stages, there were 28 (14.4%), 93 (47.7%), and 74 (37.9%) patients with TNM stage I, stage II, and stage III, respectively (Table 1).

**Table 1:** Clinical characteristics of CRC patients.

Items	CRC patients (*N* = 195)
Age (years), mean ± SD	65.7 ± 10.3
Gender, No. (%)	
Male	118 (60.5)
Female	77 (39.5)
Pathological grade, No. (%)	
Grade I	27 (13.9)
Grade II	135 (69.2)
Grade III	33 (16.9)
Tumor size (cm), median (IQR)	4.5 (3.5–5.0)
LYN metastasis, No. (%)	
Negative	121 (62.1)
Positive	74 (37.9)
T stage, No. (%)	
T1	5 (2.6)
T2	23 (11.8)
T3	165 (84.6)
T4	2 (1.0)
N stage, No. (%)	
N0	121 (62.1)
N1	50 (25.6)
N2	24 (12.3)
TNM stage, No. (%)	
Stage I	28 (14.4)
Stage II	93 (47.7)
Stage III	74 (37.9)

*CRC, colorectal cancer; SD, standard deviation; IQR, interquartile range; LYN, lymph node*.

### Comparison of SOX30 IHC Score and SOX30 mRNA Expression Between Tumor Tissue and Adjacent Tissue

**Figure 1:**
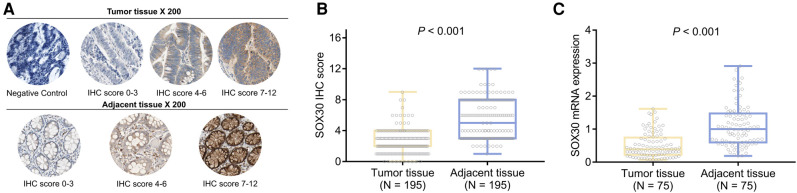
SOX30 in CRC patients. IHC images of SOX30 expression in tumor tissue and adjacent tissue (**A**); comparison of SOX30 IHC score (**B**) and SOX30 mRNA expression (**C**) between tumor tissue and adjacent tissue. SOX30, sex-determining region Y-box containing gene 30; IHC, immunohistochemistry; CRC, colorectal cancer.

SOX30 IHC score (mean ± standard deviation: 2.6 ± 1.6 vs. 5.5 ± 2.6) was decreased in tumor tissue compared to adjacent tissue (*P *< 0.001) (Figures 1A,B). Meanwhile, SOX30 mRNA expression (median (inter quartile range): 0.392 (0.221–0.744) vs. 1.000 (0.599–1.475)) was also reduced in tumor tissue compared to adjacent tissue (*P *< 0.001) (Figure 1C). In addition, antibody against SOX30 was verified by western blot, which showed that SOX30 detected by western blot was highly correlated with SOX30 detected by IHC (Supplementary Figures 1A–C).

### Correlation of Tumor SOX30 With Clinicopathological Features in CRC Patients

There were 145 CRC patients’ tumor tissue IHC score ≤3 and 50 CRC patients’ tumor tissue IHC score >3. Tumor SOX30 protein expression was negatively correlated with tumor size, LYN metastasis, T stage, N stage, and TNM stage (all *P *< 0.05); however, no correlation was found in tumor SOX30 protein expression with age, gender, or pathological grade (all *P *> 0.05). Furthermore, there were 46 CRC patients’ tumor SOX30 mRNA expression ≤0.392 and 47 CRC patients’ tumor SOX30 mRNA expression >0.392. Tumor SOX30 mRNA expression was negatively correlated with LYN metastasis, T stage, N stage, and TNM stage (all *P *< 0.05), while no correlation was found in tumor SOX30 mRNA expression with age, gender, pathological grade, or tumor size (all *P *> 0.05) (Table 2).

**Table 2:** Correlation of tumor SOX30 with clinicopathological features.

Items	SOX30 protein expression			SOX30 mRNA expression		
	Low	High	*P*-value	Low	High	*P*-value
Number of patients	145	50	0.080	46	47	0.507
Age, No. (%)						
<60 years	41 (28.3)	21 (42.0)		16 (34.8)	13 (27.7)	
≥60 years	104 (71.7)	29 (58.0)		30 (65.2)	34 (72.3)	
Gender, No. (%)			0.738			0.194
Female	56 (38.6)	21 (42.0)		13 (28.3)	20 (42.6)	
Male	89 (61.4)	29 (58.0)		33 (71.7)	27 (57.4)	
Pathological grade, No. (%)			0.892			0.852
Grade I	22 (15.2)	5 (10.0)		9 (19.6)	5 (10.6)	
Grade II	97 (66.9)	38 (76.0)		27 (58.7)	37 (78.7)	
Grade III	26 (17.9)	7 (14.0)		10 (21.7)	5 (10.6)	
Tumor size, No. (%)			0.049			0.132
<5 cm	91 (62.8)	39 (78.0)		26 (56.5)	34 (72.3)	
≥5 cm	54 (37.2)	11 (22.0)		20 (43.5)	13 (27.7)	
LYN metastasis, No. (%)			0.018			0.001
Negative	83 (57.2)	38 (76.0)		20 (43.5)	36 (76.6)	
Positive	62 (42.8)	12 (24.0)		26 (56.5)	11 (23.4)	
T stage, No. (%)			0.001			0.019
T1	2 (1.4)	3 (6.0)		0 (0.0)	1 (2.1)	
T2	12 (8.3)	11 (22.0)		1 (2.2)	6 (12.8)	
T3	129 (89.0)	36 (72.0)		44 (95.7)	40 (85.1)	
T4	2 (1.4)	0 (0.0)		1 (2.2)	0 (0.0)	
N stage, No. (%)			0.034			0.004
N0	83 (57.2)	38 (76.0)		20 (43.5)	36 (76.6)	
N1	42 (29.0)	8 (16.0)		20 (43.5)	8 (17.0)	
N2	20 (13.8)	4 (8.0)		6 (13.0)	3 (6.4)	
TNM stage, No. (%)			0.001			<0.001
Stage I	14 (9.7)	14 (28.0)		1 (2.2)	7 (14.9)	
Stage II	69 (47.6)	24 (48.0)		19 (41.3)	29 (61.7)	
Stage IIIs	62 (42.8)	12 (24.0)		26 (56.5)	11 (23.4)	

*SOX30, sex determining region Y-box family factor 30; LYN, lymph node.*

### Correlation of Tumor SOX30 With Accumulating OS in CRC Patients

**Figure 2:**
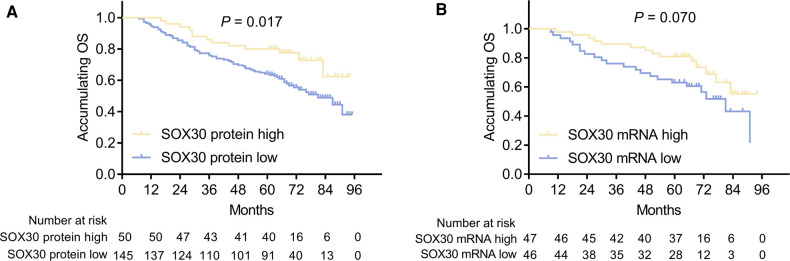
Correlation between SOX30 expression and long-term prognosis among CRC patients. Correlation of SOX30 protein expression (**A**) and SOX30 mRNA expression (**B**) with accumulating OS. SOX30, sex-determining region Y-box containing gene 30; OS, overall survival; CRC, colorectal cancer.

In order to explore the prognostic value of tumor SOX30 protein and mRNA expressions in CRC, we gathered the OS data of all CRC patients and we found that tumor SOX30 protein expression was positively correlated with accumulating OS (*P *= 0.017) (Figure 2A). However, tumor SOX30 mRNA expression was not correlated with accumulating OS (*P *= 0.070) (Figure 2B). Further subgroup analysis discovered that a positive correlation was only found in tumor SOX30 protein expression with OS in patients with TNM stage II (*P *= 0.007) (Table 3). In addition, data from The Human Protein Atlas (derived from The Cancer Genome Atlas (TCGA)) and GEPIA databases also revealed that SOX30 mRNA expression showed a tendency to be positively correlated with accumulating OS, but did not reach statistical significance (Supplementary Figures 2A,B).

**Table 3:** Correlation of tumor SOX30 with OS in subgroups categorized by TNM stage.

Subgroup	Mean OS (months)				
	SOX30 protein high	95% CI	SOX30 protein low	95% CI	*P-*value
TNM stage I	73.8	62.1–85.5	85.5	76.4–94.5	0.585
TNM stage II	89.2	82.9–95.6	72.0	64.9–79.1	0.007
TNM Stage III	55.4	39.8–71.1	57.9	49.9–65.9	0.740
	SOX30 mRNA high	95% CI	SOX30 mRNA low	95% CI	*P*-value
TNM stage I	77.8	70.5–85.1	–	–	0.705
TNM stage II	79.7	70.3–89.1	62.2	50.5–73.9	0.178
TNM Stage III	68.6	54.5–82.7	64.1	52.1–76.2	0.812

*SOX30, sex determining region Y-box family factor 30; OS, overall survival; CI, confidence interval*.

### Univariate and Multivariate Cox’s Regression Model Analysis for OS

**Figure 3:**
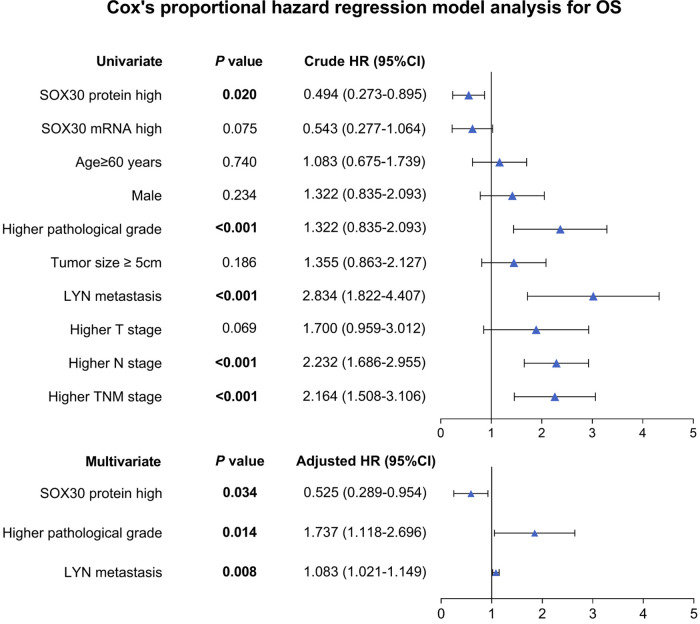
Independent factors for OS. SOX30, sex-determining region Y-box containing gene 30; LYN, lymph node; TNM, tumor node metastasis; OS, overall survival; HR, hazard ratio; CI, confidence interval.

Univariate Cox’s regression analysis showed that tumor SOX30 protein high expression was correlated with better OS (hazard ratio (HR): 0.494, *P *= 0.020); while higher pathological grade (HR: 1.322, *P *< 0.001), LYN metastasis (HR: 2.834, *P *< 0.001), higher N stage (HR: 2.232, *P *< 0.001) and higher TNM stage (HR: 2.164, *P *< 0.001) were all associated with poor OS. Moreover, multivariate Cox’s regression analysis illustrated that tumor SOX30 protein high expression was independently associated with favorable OS (HR: 0.525, *P *= 0.034); while higher pathological grade (HR: 1.737, *P *= 0.014) and LYN metastasis (HR: 1.083, *P *= 0.008) were both independently correlated with poor OS (Figure 3).

## Discussion

In our study, we discovered several interesting results: (1) SOX30 protein and mRNA expressions were both reduced in CRC tumor tissue; (2) tumor SOX30 protein expression was negatively correlated with tumor size, LYN metastasis, T stage, N stage, and TNM stage; tumor SOX30 mRNA expression was negatively correlated with LYN metastasis, T stage, N stage, and TNM stage; (3) tumor SOX30 protein expression was positively correlated with accumulating OS, furthermore, tumor SOX30 protein high expression was independently associated with favorable OS.

SOX30 is reported to be a suppressor of several cancers, which exerts its function mainly through inhibition of the Wnt/β-catenin signaling pathway and activation of p53 transcription (15, 16). In terms of SOX30 expression in cancer, a previous study illustrated that SOX30 expression is reduced in breast cancer tissue compared to adjacent tissue (11). Another interesting study also reports that SOX30 expression is particularly reduced in non-small cell lung cancer tissues compared to adjacent noncancerous tissues (10). In our study, we found that tumor SOX30 protein and mRNA expressions were both reduced in CRC tumor tissue compared to adjacent tissue, which was partly consistent with previous studies (11, 15). Possible explanations might be that SOX30 downregulation might inhibit oncogenesis-related signaling pathways in CRC, such as Wnt and β-catenin signaling pathways (11, 15, 17). Therefore, SOX30 expression was decreased in CRC tumor tissue.

Regarding the correlation of SOX30 with clinicopathological features in cancers, it has been presented that elevated SOX30 expression is associated with deficient LYN metastasis and lower TNM stage in lung cancer patients (10); a negative association has been found between SOX30 and TNM stage in bladder cancer patients (12); moreover, SOX30 is also negatively associated with tumor size in hepatocellular carcinoma patients (7). In our study, negative associations were also found in tumor SOX30 with LYN metastasis, T stage, N stage, and TNM stage in CRC patients. The possible reasons would be that: (1) SOX30 might be able to suppress proliferation and promote apoptosis of CRC cells *via* activating p53 transcription, consequently inhibiting tumor growth of CRC (7, 18). Therefore, negative associations were found in tumor SOX30 with T stage in CRC patients; (2) SOX30 might suppress CRC cell metastasis and lymph node metastasis through attenuating Wnt-signaling by modulating the transcription of β-Catenin (8, 19). Thus, negative correlations were found in SOX30 with LYN metastasis, N stage, and TNM stage in CRC patients.

With respect to the association between SOX30 and prognosis in cancers, several studies have disclosed that SOX30 is correlated with a better prognosis (9, 11, 12). For instance, previous research has presented that a positive association is found between SOX30 expression and OS in bladder cancer patients (12). Another study also illustrates that increased SOX30 expression predicts more favorable OS in ovarian cancer patients (9). In the present study, a positive correlation was observed in tumor SOX30 expression with accumulating OS in total CRC patients, and in the TNM stage II subgroup; more notably, multivariate Cox’s proportional hazards regression analysis presented that tumor SOX30 protein high expression was independently associated with favorable OS, which was partly consistent with previous studies (9, 11, 12). Potential explanations could be that: (1) SOX30 could decrease chemoresistance in CRC cells by promoting p53 transcriptional activation, thereby enhancing the effect of CRC therapy, which could directly improve the prognosis of CRC (7, 20); (2) tumor SOX30 was correlated with better tumor clinical features (above-mentioned), which could indirectly lead to better prognosis in CRC. Therefore, SOX30 was positively correlated with CRC prognosis.

There existed several limitations in our study: (1) we only recruited the patients who were diagnosed with primary CRC, the clinical value of SOX30 in patients with secondary CRC could be investigated in the future; (2) the present study was a retrospective study, which might exist a series of compound factors and bias; however, we tried to use multivariate Cox’s regression model analysis to eliminate partial interfering factors; (3) the role of SOX30 in the regulatory mechanism of CRC could be investigated and further to explore the therapeutic value of SOX30 in CRC.

In conclusion, tumor SOX30 negatively associates with LYN metastasis, T stage, N stage, and TNM stage while positively relates to accumulating OS in CRC patients, suggesting SOX30 might be a possible biomarker of progression and prognosis of CRC.

## Data Availability

The original contributions presented in the study are included in the article/Supplementary Material, further inquiries can be directed to the corresponding author/s.
