# Supplementary material for: SOX30 Overexpression Reflects Tumor Invasive Degree, Lymph Node Metastasis and Predicts Better Survival in Colorectal Cancer Patients: A Long-Term Follow-Up Cohort Study
Source: Front Surg. 2022 Jun 28;9:898952. doi: 10.3389/fsurg.2022.898952 (PMC9273907; doi:10.3389/fsurg.2022.898952)
Supplement: Supplementary file 1 [file Data_Sheet_1_v1.pdf]

Supplementary Materials

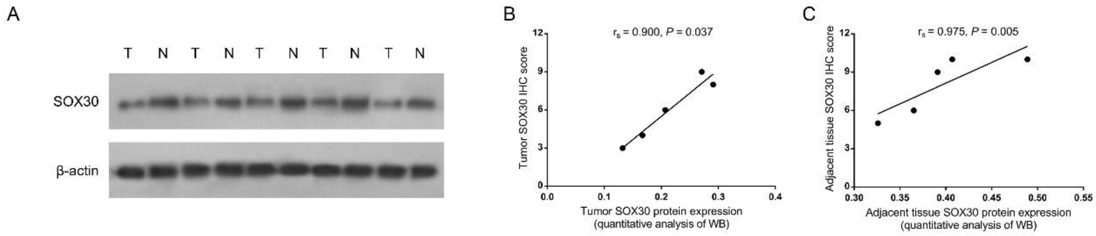

Supplementary figure 1. Verification of antibody against SOX30 by western blot. SOX30 detected by western blot in CRC and adjacent tissues. T represented tumor tissue and N represented adjacent tissues (**A**). Correlation of SOX30 protein expression detected by western blot and SOX30 IHC score in tumor tissues (**B**) and adjacent tissues (**C**).

A

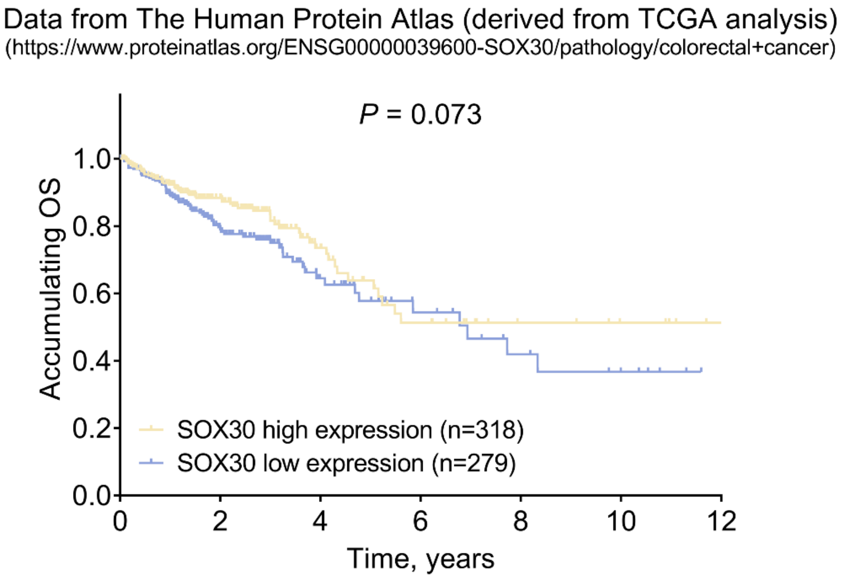

B

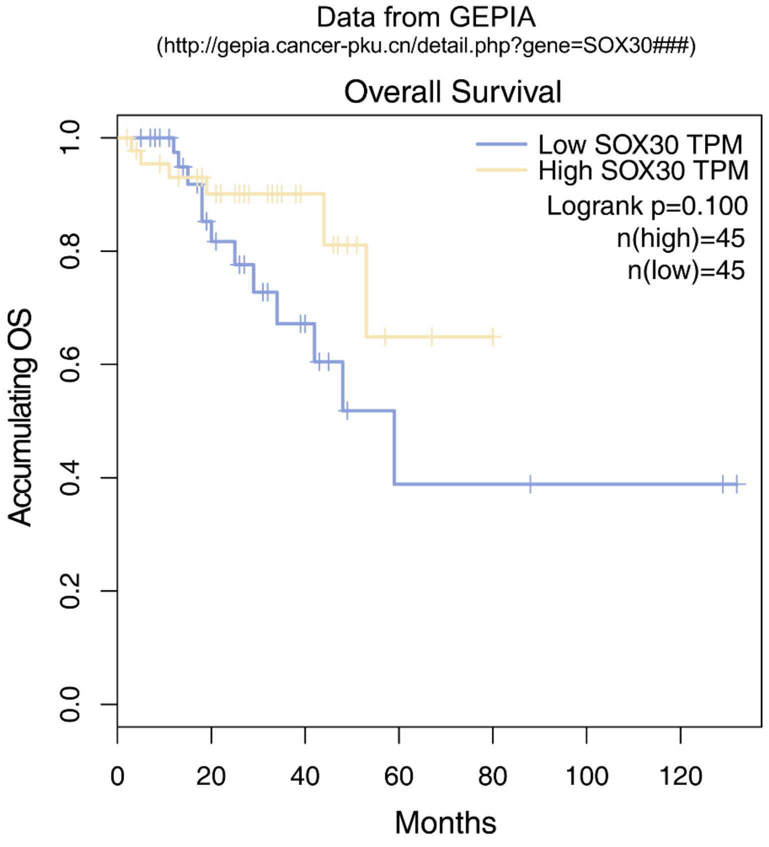

Supplementary figure 2. Prognostic value of SOX30 mRNA expression verified by data from public database. Correlation between SOX30 mRNA expression and accumulating OS by data from The Human Protein Atlas (derived from The Cancer Genome Atlas (TCGA) (**A**) and GEPIA (**B**).
